# Pituitary Adenylate Cyclase Activating Polypeptide Modulates Catecholamine Storage and Exocytosis in PC12 Cells

**DOI:** 10.1371/journal.pone.0091132

**Published:** 2014-03-06

**Authors:** Yan Dong, Gang Ning, Andrew G. Ewing, Michael L. Heien

**Affiliations:** 1 Department of Neurosurgery, Changzheng Hospital, Second Military Medical University, Shanghai, China; 2 Department of Chemistry, The Pennsylvania State University, University Park, Pennsylvania, United States of America; 3 Huck Institutes of the Life Sciences, The Pennsylvania State University, University Park, Pennsylvania, United States of America; 4 Department of Neural and Behavioral Sciences, The Pennsylvania State University College of Medicine, Hershey, Pennsylvania, United States of America; 5 Department of Chemistry, University of Gothenburg, Göteborg, Sweden; 6 Department of Chemistry and Biochemistry, The University of Arizona, Tucson, Arizona, United States of America; University of Rouen, France

## Abstract

A number of efforts have been made to understand how pituitary adenylate cyclase activating polypeptide (PACAP) functions as a neurotrophic and neuroprotective factor in Parkinson’s disease (PD). Recently its effects on neurotransmission and underlying mechanisms have generated interest. In the present study, we investigate the effects of PACAP on catecholamine storage and secretion in PC12 cells with amperometry and transmission electron microscopy (TEM). PACAP increases quantal release induced by high K^+^ without significantly regulating the frequency of vesicle fusion events. TEM data indicate that the increased volume of the vesicle is mainly the result of enlargement of the fluidic space around the dense core. Moreover, the number of docked vesicles isn’t modulated by PACAP. When cells are acutely treated with L-DOPA, the vesicular volume and quantal release both increase dramatically. It is likely that the characteristics of amperometric spikes from L-DOPA treated cells are associated with increased volume of individual vesicles rather than a direct effect on the mechanics of exocytosis. Treatment with PACAP versus L-DOPA results in different profiles of the dynamics of exocytosis. Release via the fusion pore prior to full exocytosis was observed with the same frequency following treatment with PACAP and L-DOPA. However, release events have a shorter duration and higher average current after PACAP treatment compared to L-DOPA. Furthermore, PACAP reduced the proportion of spikes having rapid decay time and shortened the decay time of both fast and slow spikes. In contrast, the distributions of the amperometric spike decay for both fast and slow spikes were shifted to longer time following L-DOPA treatment. Compared to L-DOPA, PACAP may produce multiple favorable effects on dopaminergic neurons, including protecting dopaminergic neurons against neurodegeneration and potentially regulating dopamine storage and release, making it a promising therapeutic agent for the treatment of PD.

## Introduction

Parkinson’s disease (PD) is a chronic neurodegenerative disorder characterized by progressive loss of dopaminergic neurons in the substantia nigra pars commpacta (SNc), coupled with the presence of intracellular proteinaceous inclusions known as Lewy bodies [1]. Depletion of dopamine in the nigrostriatal system attributes to the motor disturbances, vegetative, sensory and psychopathological symptoms in PD patients. Current available symptomatic therapy is primarily based on dopamine modulation or substitution strategies, which fail to prevent, delay or stop the process of PD [2]–[3]. The progressive degeneration of dopaminergic neurons precedes onset of motor symptoms, with approximately 70% of neurons in the SNc being lost prior to the appearance of motor features [4]. Hence, it is of great significance to diagnose PD during the early stage of the disease and subsequently prevent dopaminergic neurons in the SNc from degeneration in the management of PD. Over the past two decades, comprehensive understanding of the mechanisms responsible for cell death in PD has rendered the identification of putative neuroprotective and restorative treatment [5]–[6].

Pituitary adenylate cyclase activating polypeptide (PACAP) is a 38 amino acid neuropeptide, which is first isolated from ovine hypothalamus and now known to regulate the development, maintenance, function and plasticity of the nervous system, providing neuroprotective and neurotrophic support [7]–[10]. PACAP and its receptors are present in the developing and adult rat mesencephalon, and both PACAP binding sites and their mRNA have been identified in the SNc [11]–[13]. In mesencephalic cultures, PACAP increased the number of tyrosine hydroxylase (TH) immunoreactive neurons, and enhances dopamine uptake. Moreover, pretreatment of the mesencephalic cultures with PACAP protects dopaminergic neurons against 6-OHDA-induced neurotoxicity. Recently, more and more evidence verifies the protective effects of PACAP in PD model in vivo [14]–[21]. Apart from being a neurotrophic and neuroprotective factor, PACAP also acts as a modulator and neurotransmitter to regulate neurotransmission. PACAP can act as a potent modulator of glutamatergic and nicotinic signaling [22]–[24]. Additionally, PACAP potentiates catecholamine release. PACAP induces catecholamine release from adrenal chromaffin cells, sympathetic nucleus neurons and neurosecretory cells, respectively, by elevating intracellular Ca^2+^ concentration [25]–[27]. These results suggest that PACAP plays an important role in the modulation of synaptic transmission. However, the precise mechanism has not yet been clarified.

The introduction of levodopa is a milestone in the treatment of PD. Although levodopa remains the most widely employed and most effective antiparkinsonian drug and provides extraordinarily clinical benefits in reducing the symptoms of PD, concern that lepodopa might actually hasten neurodegeneration in PD patients due to its cytotoxic effect has been widely raised [28]–[31]. PACAP is a neuropeptide with 38 amino acid residues, which can penetrate the blood-brain barriers. Compared to levodopa, PACAP might produce multiple favorable effects on dopaminergic neurons, including protecting dopaminergic neurons against various damages and potentially regulating dopamine release, rendering it to be a promising therapeutic agent in Parkinson's disease. A successful PACAP therapy for PD will require an in-depth molecular and integrative understanding of the impact of PACAP on physiological and pathological process that plays in dopaminergic neurons.

Catecholamine is stored in LDCVs in chromaffin cells and PC12 cells, while they are packaged in the small synaptic vesicles in dopaminergic neurons in SNc. Synaptic vesicle fusion in cultured ventral midbrain dopaminergic neurons typically releases ∼3000 dopamine molecules per quantum [32], which is more than 2–3 orders of magnitude smaller than in chromaffin cells and PC12 cells. Furthermore, the fast quantal release cannot be recorded in vivo or in striatal slices because it is not possible to position a carbon fiber electrode in direct contact with one or several intact release sites [33]. Owing to lack of effective means to investigate quantal release in midbrain dopaminergic neurons, PC12 cells and genetically modified PC12 cell lines are extensively applied to study the synthesis and release of catecholamines. In the present study, we treated PC12 cells for 3 days with 100 nm PACAP and triggered excytosis in PC12 cells with 100 nM KCl. The effects of PACAP on catecholamine storage and quantal secretion in PC12 cells were determined with amperometry and transmission electron microscopy (TEM). PACAP increases quantal release and vesicular volume. TEM data indicate that the volume of fluidic space around the vesicular dense core, or halo, is the main player in the enlargement of vesicle volume. PACAP increases neurotransmitter flux and dilation of the fusion pore. By comparison, L-DOPA also increases vesicular volume dramatically, but displays different kinetics for fusion events than those observed following PACAP.

## Materials and Methods

### Cell Culture

Stock PC12 cells were generously provided by Dr. Dave Sulzer (Columbia University) and maintained as described previously [34]. In brief, PC12 cells, plated on mouse collagen IV-coated culture dishes (Becton Dickinson, Bedford, MA) in RPMI-1640 medium supplemented with horse serum, fetal bovine serum and penicillin/streptomycin, were kept in a 7% CO_2_ atmosphere at 37°C. On the second day, 100 nM PACAP (Peninsula Laboratories, Belmont, CA) was added to the cultures. The medium was changed and PACAP replenished every 24 h. Experiments were carried out after 4 days of culture.

### Electrochemical Recording

Amperometric recordings were made as described previously [35]. Briefly, electrodes were held at +0.65 V vs a Ag/AgCl reference electrode (World Precision Instruments, Inc., Sarasota, FL, USA) using a commercially available patch-clamp instrument (Axopatch 200B; MDS Analytical Technologies, Sunnyvale, CA, USA). The amplifier output was filtered at 2 kHz using a four-pole low-pass Bessel filter and digitized at 5 kHz. Data were displayed in real time and stored in the computer with no further filtering. Exocytosis was stimulated at 40-s intervals with a 5-s, 20-psi pulse (Picospritzer II; General Valve, Fairfield, NJ, USA) of physiological saline with elevated potassium (100 mM KCl). The concentration of NaCl in the elevated KCl solution was adjusted to maintain isotonicity. All experiments were performed at 37±1°C. Exocytotic spikes were identified (> five times the noise), and the spike characteristics – area (pC), *t_1/2_* (ms), and *I_max_* (pA) – were determined using a multipass algorithm described previously [36]. The number of molecules detected was calculated from the charge under each amperometric spike by use of the relationship, Q = nNF, where Q is the charge of each current transient, N is the number of moles, F is Faraday’s constant (96,485 C/equiv), and n is the number of electrons transferred per oxidized molecule (this was assumed to be 2 for catecholamines). Signals were designated as spikes if their *I_max_* values were five times the noise (typically 0.6 pA) of a 1-s portion of stable baseline recorded before the first stimulation. All peaks that were identified by the program (Mini Analysis; Synaptosoft Inc, Decatur, GA, USA) were inspected visually, and overlapping peaks were excluded manually from the datasets.

### Transmission Electron Microscopy

PC12 cells were rinsed with RPMI-1640 medium without serum and detached from the flasks. Single cell suspensions were transferred to Microfuge tubes and pelleted at 100×*g* for 10 min. Cell pellets were fixed with an ice-cold fixative containing 2% glutaraldehyde in 0.1 M phosphate buffer, pH 7.4 at room temperature for 1 h, and then incubated overnight at 4°C. The cells were post-fixed in 1% OsO_4_ for 1 h and dehydrated by serial treatment in solutions of graded ethanol and embedded in Eponite 12. The areas of interest were selected under a dissecting microscope and 80-nm-thick sections were produced with an ultramicrotome (Reichart Microscopy, Depew, NY, USA). Sections were contrast enhanced with uranyl acetate and lead citrate and examined with a JEOL JEM 1200 EXII transmission electron microscope (JEOL, Peabody, MA, USA) at 80 kV.

Quantitative analysis of vesicle structures was performed using Image J 1.37v (Wayne Rasband, NIH, Bethesda, MD, USA). Transmission electron microscopy images were imported into this software and the limiting membrane of each vesicle as well as the perimeter of its dense core were traced. Once each object was inscribed, Image J determined its diameter (the average distance of the major and minor axis on the initial trace). Only vesicles in which a dense core could be clearly identified were measured.

### Statistics

To ensure that cells with a large number of events or vesicles would not be overrepresented within a treatment group, mean cellular values were included in the analysis of quantal size, amplitude, half-width and TEM data. Data sets were tested for significant differences using *t*-test or ANOVA (Prism; Graph-Pad Software, Inc., La Jolla, CA, USA). Results were considered significant if associated *p*-values were less than 0.05. All values are reported as the mean ± SEM.

## Results

### PACAP Increases Quantal Release Amount

**Figure 1:**
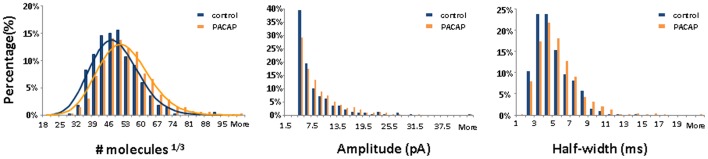
Histograms of the cube root of quantal size, amplitude and half-width for control and PACAP-treated PC12 cells (n = 482 events for control cells, n = 1436 events for PACAP-treated cells). Distribution of cube root transforms of the quantal sizes can be fitted with one Gaussian function in both control and PACAP-treated cells.

PACAP often co-localizes with classical neurotransmitters, exerting both rapid-onset modulatory influences on synapses and gradual neurotrophic influences on neuronal survival [22], [37]. Microarray analyses indicate that the expression of some neuroendocrine-specific genes, such as *Kcna2* and *Snap25*, is time-consuming [38]. To ensure that the effects of PACAP on the PC12 cells reached full equilibrium, PC12 cells were exposed to 100 nM PACAP for 3 days. Of note, no neurosecretion was seen unless PC12 cells were stimulated by high K^+^. In control PC12 cells, an average of 107,600±7,200 molecules were released from each vesicle following stimulation with high K^+^-containing buffer. PACAP treatment increased the quantal size of evoked exocytosis to 147,100±16,000 molecules (Table 1). PACAP did not significantly alter the distribution of quantal size, as only one population of cube root transforms of quantal size is present in both control and PACAP-treated PC12 cells (Figure 1). In addition, distributions of spike amplitude and halfwidth were quite similar (Figure 1). Both were not significantly affected by treatment with 100 nm PACAP (Table 1).

**Table 1:** Summary of the shape characteristics of individual release events from control, PACAP-treated, and L-DOPA-treated cells.

	PACAP		L-DOPA	
	Control (n = 13)	PACAP (n = 16)	Control (n = 35)	L-DOPA (n = 27)
*I_max_* (pA)	6.4±0.58	8.0±0.69	8.19±3.62	9.84±3.26
Quantal size (number of molecules)	107,600±7,200	147,100±16,000*	78,014±17,611	127,042±50,220**
Half-width (ms)	4.3±0.38	4.5±0.46	2.49±0.68	3.44±1.19**

*p<0.05 vs. control,

**p<0.01 vs. control.

### PACAP Elevates the Vesicular and Halo Volume

**Figure 2:**
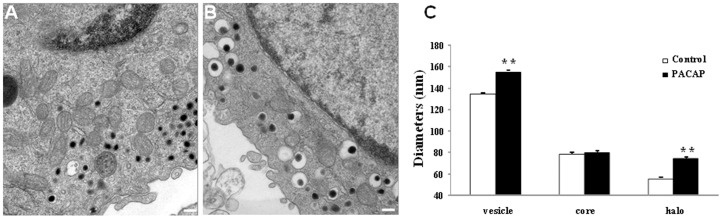
Representative TEM images of (A) control and (B) PACAP-treated cells. Large dense core vesicles are distributed near and far from the plasma membranes. A portion of the nucleus can be seen in the cells. Scale bars = 200 nm. (C) Mean vesicle sizes of control and PACAP-treated cells (n = 17 cells from control group, n = 24 cells for PACAP-treated group; **p<0.01 vs. control cells, *t-*test).

Previous work has shown that the increase in quantal size is often associated with the enlargement of vesicle diameter [32], [39]. Hence, we analyzed large dense core vesicles (LDCVs) from PACAP-treated cells with transmission electron microscopy (TEM). Representative TEM images from single PC12 cells are shown in Figure 2. For all cells examined, LDCVs could be readily observed throughout the cytoplasm. Additionally, for all vesicles analyzed, a limiting membrane could be clearly discerned from the dense core. The mean diameter of the outer limiting membrane for LDCVs from control cells was 134±4 nm. This value increased by 16% to 155±5 nm when PC12 cells were treated with 100 nM PACAP for 3 days (p<0.01 vs. control, *t-*test). Furthermore, similar measurements were made of the diameter of the vesicular dense core. The corresponding diameter of the fluidic volume (halo) around the dense core was calculated by subtracting the diameter of the dense core from that of the outer limiting membrane. A significant elevation of the diameter of the halo was observed in PACAP-treated PC12 cells (p<0.01), whereas PACAP did not influence the diameter of the dense core (p>0.05, Figure 2). These results indicate that PACAP increases the volume of large dense core vesicle mainly by expanding the volume of the halo.

### PACAP Changes the Dynamics of Fusion Events

**Figure 3:**
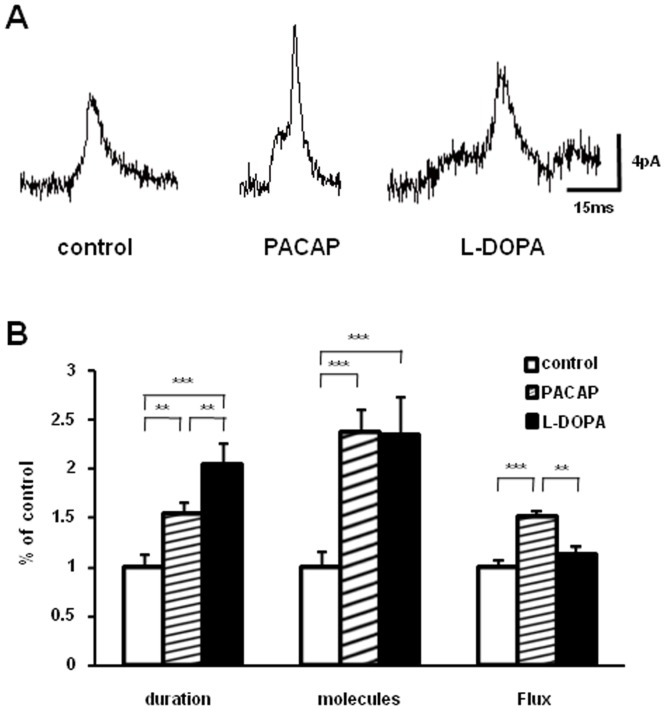
Representative amperometric foot current transients (A) and summary of foot duration, foot quantal size and mean catecholamine flux (B). Flux was computed as foot area divided by duration. Error bars represent mean ± SEM (control, 93 events; PACAP, 146 events and L-DOPA, 52 events). *** p<0.001 and ** p<0.01 vs. control, respectively (ANOVA test).

To address the effect of PACAP on vesicle fusion, we examined several spike parameters relevant to vesicle fusion events. Because PACAP increases the vesicle volume, we argued that changed vesicle volume might alter the kinetics of vesicle fusion. As L-DOPA has also been shown to increase the vesicle volume, we treated PC12 cells with 100 µM L-DOPA at 37°C for 90 min and examined the kinetics of fusion. To compare the effect of PACAP and L-DOPA directly, ratio values for the spike characteristics foot, rise time, and decay time are presented (Figure 3).

Amperometric transients from exocytosis are often preceded by a small amperometric current, called the “foot” of the spike, indicating the existence of a dynamic fusion pore prior to full exocytosis. The number of molecules released during the prespike foot, the length of the foot and the average current (foot area divided by foot duration) have been determined here for each amperometric spike recorded. Values have been pooled and plotted (Figure 3). PACAP and L-DOPA both equally increase the number of molecules released from the foot. However, when compared to L-DOPA, release events from PACAP-treated cells had a shorter duration with an average current that was elevated, corresponding to a higher flux of molecules from the fusion pore. In contrast, L-DOPA did not influence the average current when compared to the control.

**Figure 4:**
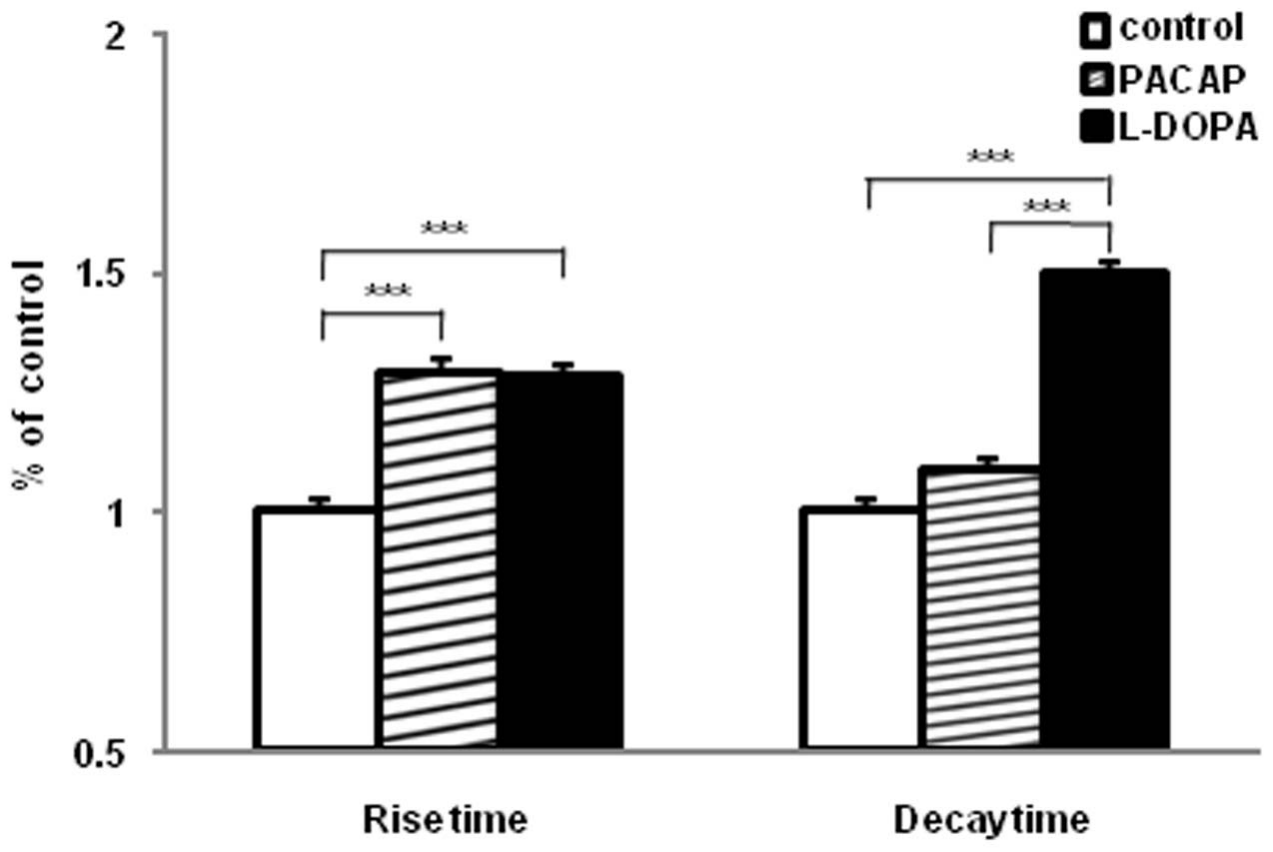
Summary of rise time and decay time in PACAP and L-DOPA treated cells. Error bars represent mean ± SEM (control, 1767 events; PACAP, 1436 events and L-DOPA, 2246 events). Significance: *** p<0.001 vs. control, respectively (ANOVA test).

**Figure 5:**
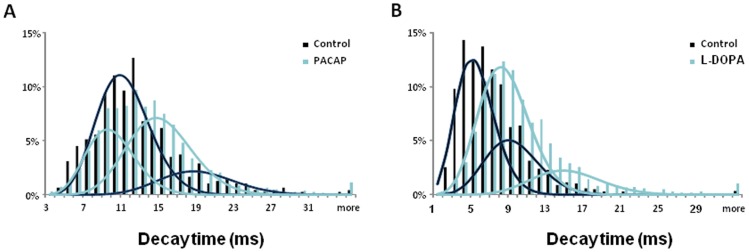
Distribution of the decay time in (A) PACAP and (B) L-DOPA treated PC12 cells. Histograms indicate the existence of two populations of spikes that can be well fitted with two Gaussian functions. PACAP reduces the proportion of rapid spikes of the decay time. Moreover, PACAP shortens the decay time of both fast and slow spikes. In contrast, distributions of decay time of both fast and slow spikes are shifted to the right by the treatment of L-DOPA.

The foot current is thought to terminate by fusion pore dilation and further vesicle distention, during which neurotransmitter rapidly escapes from the vesicle producing a spike of current at the electrode. Following both PACAP and L-DOPA the rise time of the amperometric current increased approximately 30%. Application of L-DOPA produced a 50% longer decay time for the amperometric spikes and application of PACAP did not significantly alter decay time when compared to control (Figure 4). It is possible that the difference in the decay time between PACAP- and L-DOPA-treated PC12 cells indicates the existence of two populations of spikes that can be fit with two Gaussian functions than with only one (correlation coefficients are 0.9135 for 1 and 0.9231 for 2 Gaussian fits in control cells; correlation coefficients are 0.9135 for 1 and 0.9833 for 2 Gaussian fits in PACAP-treated cells). This is shown in Figure 5. Examining these distributions, it is evident that PACAP increased the number of events with slower decay time, but the average decay time was faster for both distributions. In contrast, the distributions of the amperometric spike decay for both fast and slow spikes were shifted to longer time by the treatment of L-DOPA.

### PACAP does not Significantly Regulate the Frequency of Vesicle Fusion Events

The release of neurotransmitters from a secretory vesicle is an extremely rapid process, which is part of a much slower, complex ensemble of processes, known as the vesicle cycle. To investigate any underlying involvement of PACAP in the process of the vesicle cycle, we investigated the frequency of amperometric spikes evoked by high potassium and distribution of LDCVs with TEM following application of PACAP. Cells were stimulated with a 5-s application of 100 mM KCl three times with 40-s intervals between the stimuli. The frequency of spikes was determined simply by dividing the total number of spikes by the time elapsed from the occurrence of the first spike to the occurrence of the last spike during the first stimulation. PACAP did not significantly alter the frequency of vesicle fusion events (55±8/min for control cells; n = 13, 83±20/min for PACAP-treated cells; n = 16, *p = *0.20). Moreover, the distributions of LDCVs in control and PACAP-treated cells were approximately the same. We assumed the vesicles located within 50 nm from the plasmalemmal membrane were morphologically docked vesicles that were potentially available for secretory fusion. In control cells, 35% of the vesicles observed were docked (103 of 293), whereas in PACAP-treated cells 30% were docked (108 of 361), which is not significantly different (Fisher’s exact test, *p = *0.18).

## Discussion

We show that PC12 cells exposed to 100 nM PACAP for 3 days demonstrate an increased quantal size when compared with control cells. Existing data demonstrate that PACAP can act as a secretagogue to elicit both short-term (several minutes) and long-term (minutes to hours) catecholamine secretion in chromaffin cells [40]–[42], which constitutes an important step in stress responses. Likewise, PACAP can evoke distinct immediate and long-lasting effect on catecholamine secretion in PC12 cells [43]–[45]. In this study, although PC12 cells were treated with 100 nm PACAP for 3 days, no neurosecretion was seen unless they are stimulated by high K^+^. Hence exocytosis observed in this experiment is evoked by high K^+^ through membrane depolarization and opening of VGCCs, rather than by PACAP. Our result is consistent with more acute experiments where 10 nM PACAP puffed onto cells was shown to cause more catecholamines to be released from vesicles in chromaffin cells [46]. In the earlier work, the short exposure of the chromaffin cells to PACAP makes it unlikely that the PACAP-induced variation in release is caused by changes in gene expression in these cells. One might speculate that PACAP causes a rapid elevation of intracellular cAMP favoring granule aggregation or vesicle-vesicle combination prior to fusion with cell membrane, thus leading to increased quantal release. In the current study, however, long-term treatment of PACAP appears likely to trigger changes in gene transcription related to the catecholamine biosynthesis. Earlier work has indicated that PACAP activates PAC1 receptors increasing Tyrosine Hydroxylase (TH) gene transcription through a PKA-dependent pathway [47]–[49] and induces phenolethanolamine N-methyltransferase (PNMT) gene expression through activation of cAMP-PKA and extracellular Ca^2+^ signaling mechanisms [50]. Furthermore, PACAP increases the expression of vesicular monoamine transporter VMAT, a protein responsible for packaging monoamine into secretory vesicles [15], [51]. In addition to increasing quantal size, TEM measurements demonstrate that PACAP enlarges both vesicle and halo volumes. The findings are consistent with previous data showing that increased quantal size is accompanied by correspondingly enlarged vesicle volume [33]–[39]. We argue that PACAP might enhance the transport of catecholamines into the vesicles via VMAT, leading to an increase halo volume and quantal release. Moreover, another possible mechanism for the increased quantal size and vesicle volume is that PACAP elevates the synthesis of catecholamines. Further evidence is needed to clarify the exact mechanisms of how PACAP increase the quantal release and vesicle volume in PC12 cell.

The formation of the fusion pore is a key step in exocytosis. We monitored the small current that is often observed as a plateau immediately prior to the full exocytosis event, the amperoetric “foot” (release via the open fusion pore) and examined the lifetimes of these feet to determine whether PACAP influences the stability of the fusion pore. Our data demonstrate that PACAP causes an increased foot duration and more molecules of neurotransmitter are released via the fusion pore during these events. It appears that in PACAP-treated cells, vesicles form more stable fusion pores.

Previous data demonstrate that the change of vesicle volume affects the characteristics of amperometric spikes [35]. In this study, we show that PACAP enlarges the volume of vesicle. Beside the increased vesicle volume, other factors might contribute to the effect of PACAP on the characteristics of amperometric spikes. Application of L-DOPA, the synthetic precursor of dopamine, increases both quantal size and vesicular volume dramatically in PC12 cells [39] Changes to the characteristics of amperometric spikes from L-DOPA treated cells are likely associated with increased vesicle volume rather than any direct effect on exocytosis itself [52]. Moreover, 100 nM PACAP and 100 µM L-DOPA caused a similar increase in the mean diameter of the outer limiting membrane for LDCVs in PC12 cells (15.7% for PACAP; 21.5% for L-DOPA) [53]. In the present experiments, our results show that L-DOPA increased both the foot duration and the amount of neurotransmitter released through the exocytotic fusion pore. This is consistent with previously published work [35]. After incubation with either PACAP or L-DOPA, most of the vesicular volume increase is in the vesicular halo, where the dense core swells relatively little [39]. If release via the fusion pore is assumed to be mainly from the halo of the vesicle, then the excess catecholamine in the halo of the vesicles should increase the amount of neurotransmitter released through the exocytotic fusion pore before full exocytosis as well as the total amount released (quantal size). Although both PACAP and L-DOPA appear to stabilize fusion pore formation, the difference of fusion pore lifetime in PACAP- and L-DOPA-treated cells is significant. Vesicles in PACAP-treated cells form fusion pores with a shorter foot duration and a faster neurotransmitter flux compared to that of L-DOPA, suggesting that PACAP might influence the structure of the fusion pore as well as vesicle size. One possibility is that PACAP increases the diameter of the fusion pore, but there is no direct evidence for this. Several interesting studies on the effects of SNARE protein manipulation on the flux through the fusion pore have been published [54]–[56]. cDNA array data indicate that PACAP enhances expression of synaptotagmin IV and V, as well as SNAP-25 in PC12 cells and primary cultured sympathetic neurons[57]–[59]. Further investigation is needed to verify whether SNARE complexes and synaptotagmins are involved in the mechanism of PACAP on formation of the fusion pore.

In classical models of vesicle exocytosis, fusion pores expand to completely merge with the plasma membrane, leading to the complete release of the luminal contents. However, vesicle exocytosis can utilize alternative modes in which the fusion pore either abruptly closes (kiss-and-run) or in which the fusion pore dilates but subsequently recloses, called cavicapture. These transient modes of vesicle exocytosis lead to the partial release of luminal contents depending on the sizes and diffusibility of the cargo [60]–[61]. This could lead to “slow” versus “fast” modes of exocytosis in addition to the altered amounts released. In the present work, biomodal distributions of decay time were presented in control cells similar to that reported before [62]. Our data and others reach the same conclusion, showing that LDCV volume follows a Gaussian distribution [63]. Therefore, it is possible that the two populations of amperometric spikes correspond to two modes of vesicle fusion with different rates of fusion pore dilation rather than two groups of vesicles. Our data demonstrate that the distribution of decay time of both fast and slow spikes is shifted to longer times by the treatment of L-DOPA owing to increased vesicular volume. Considering only the effect of vesicular size, one would expect the distribution of decay time in PACAP-treated cells to be similar to the trend observed in L-DOPA-treated cells. In contrast, a significant fraction of the fast spikes have been transformed into slow spikes by treatment with PACAP. Data from Darchen’s group indicate that fast and slow fusion events employ different machineries [62], in which SNARE proteins have a key role in membrane fusion. To date, little work has been done to illustrate whether PACAP regulates SNARE complex assembling and structural transition, more experiments are necessary to explain the machinery involved in the effect of PACAP on the rate of exocytosis.

In this study, we demonstrate that PACAP increases quantal release induced by high K^+^ and vesicular volume, without significantly regulating the frequency of vesicle fusion events. Also, we examine the effects of PACAP and L-DOPA on exocytosis in PC12 cells. Despite both PACAP and L-DOPA appear to stabilize fusion pore formation, different dynamics of fusion pores in PACAP- and L-DOPA-treated cells are observed. Furthermore, PACAP might regulate the transformation of fast fusion events into slow fusion events, without similar transformation seen in L-DOPA-treated PC12 cells. PACAP might affect the structures associated with exocytosis as well as vesicle size, while the effect of L-DOPA on exocytosis is likely attributed to increased vesicle volume. Due to its multiple putative influences on dopaminergic neurons, PACAP might not only provide dopamine modulation, but also render potential neuroprotective and restorative therapy for PD patients.
